# Integrating random walk with restart and k-Nearest Neighbor to identify novel circRNA-disease association

**DOI:** 10.1038/s41598-020-59040-0

**Published:** 2020-02-06

**Authors:** Xiujuan Lei, Chen Bian

**Affiliations:** School of Computer Science, Shaanxi Normal University, Xi’an, 710119 China

**Keywords:** Computational models, Computational models, Computational models, Data processing, Data processing

## Abstract

CircRNA is a special type of non-coding RNA, which is closely related to the occurrence and development of many complex human diseases. However, it is time-consuming and expensive to determine the circRNA-disease associations through experimental methods. Therefore, based on the existing databases, we propose a method named RWRKNN, which integrates the random walk with restart (RWR) and k-nearest neighbors (KNN) to predict the associations between circRNAs and diseases. Specifically, we apply RWR algorithm on weighting features with global network topology information, and employ KNN to classify based on features. Finally, the prediction scores of each circRNA-disease pair are obtained. As demonstrated by leave-one-out, 5-fold cross-validation and 10-fold cross-validation, RWRKNN achieves AUC values of 0.9297, 0.9333 and 0.9261, respectively. And case studies show that the circRNA-disease associations predicted by RWRKNN can be successfully demonstrated. In conclusion, RWRKNN is a useful method for predicting circRNA-disease associations.

## Introduction

CircRNA, as a star molecule in the recent years, is a kind of non-coding endogenous RNA with single-stranded, closed and circular structure^1,2^. Unlike the linear RNA, circRNA is the result of “back-splice” or derived from linear RNA. Hence, they lack 5′-3′ ends representing the RNA transcription’s start and stop^3–6^. The first circRNA was discovered by electron microscopy in RNA viruses^7^ and afterwards in eukaryotic cells^8^. Unfortunately, researchers regarded circRNA initially as a by-product of abnormal splicing without regulatory potential. Thus, circRNA did not attract much scientific attention^9^.

With the increasing researches on circRNAs, lots of circRNAs have been found in viruses, animals and plants^6,10–12^. So far, circRNA has been confirmed to regulate multiple major biological processes, like cell invasion, proliferation as well as apoptosis^13,14^. And circRNA is an important part in process of transcription^15^, mRNA splicing^16^, RNA translation and decay^17^. Thus, the regulatory mechanism of circRNA is closely related to the occurrence of disease, which was identified by advanced biotechnology. For instance, the expression level of hsa_circ_0001982 in breast cancer tissues is significantly high^18^. In addition, there are some circRNAs (Hsa_circ_0014717^19^, CircMTO1^20^, Circ-PRKCI^21^) that act as miRNA’s sponge to regulate tumorigenesis. Therefore, it can provide new ideas for the treatment of diseases with acquisition and utilization of information related to circRNAs and diseases.

In recent years, some circRNA-disease related databases have also been proposed to further investigate the associations between circRNAs and diseases, involving CircR2Disease^22^, circRNADisease^23^ and Circ2Disease^24^. The effective calculation methods based on these databases will effectively reduce the time consumption caused by the methods in biological experiments. Thus, it is urgent to use computational methods for exploring disease-related circRNA. Fan *et al*.^25^ raised a similarity-based method with KATZ measure called KATZHCDA on a heterogeneous network. Yan *et al*.^26^ advanced a kernel-based method with regularized least squares. Lei *et al*.^27^ proposed a path-weighted method (PWCDA) integrating disease functional similarities and circRNA semantic similarities. Xiao *et al*.^28^ put forward a model (MRLDC) using a weighted manifold regularized-based algorithm. Wei *et al*.^29^ proposed a factorization Machine (FM) based method called iCircDA-MF using matrix factorization. Zhao *et al*.^30^ developed a method (IBNPKATZ) integrating the KATZ measure and bipartite network projection. Zhang *et al*.^31^ proposed a label propagation method (CD-LNLP) based on linear neighborhood. However, these methods above rely on the information of circRNA-disease, circRNAs or diseases, and the number of datasets is relatively limited. Therefore, it is not very suitable to discover the relationship of new diseases and novel circRNAs. To solve the problem further, Deng *et al*.^32^ proposed a KATZ-based method (KATZCPDA) integrating the information of circRNAs, diseases and proteins. Due to bioinformatics analysis of protein information, KATZCPDA could predict potential association that cannot be inferred when only using information of circRNAs and diseases.

**Figure 1:**
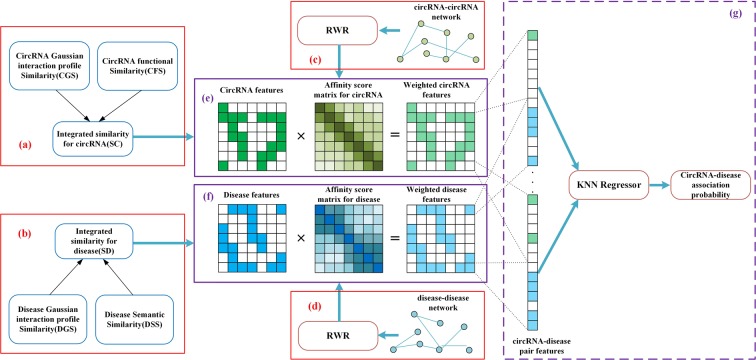
The flowchart of the computational method RWRKNN.

Inspired by Lee *et al*.^33^, a model weighting the features of circRNAs and diseases in the global network topology is put forward. In this work, all features of circRNA-disease pairs are weighted using the random walk with restart (RWR) algorithm. Firstly, we construct circRNA-disease associations, and calculate circRNA functional similarity, Gaussian interaction profile (GIP) kernel similarity of circRNAs, disease semantic similarity and GIP kernel similarity of diseases. Secondly, based on these similarities, we further construct two matrixes, *i.e*., circRNA-circRNA matrix, disease-disease matrix. Next, the RWR is performed on all nodes in circRNA-circRNA matrix and disease-disease matrix respectively. With affinity scores of all circRNA and disease nodes from RWR, features of circRNAs (diseases) consisting of integrated circRNA (disease) similarity are weighted. In the end, negative circRNA-disease pairs are selected randomly and a k-Nearest Neighbor (KNN) model get trained with weighted features (See Fig. 1).

## Results

### Performance evaluation

**Figure 2:**
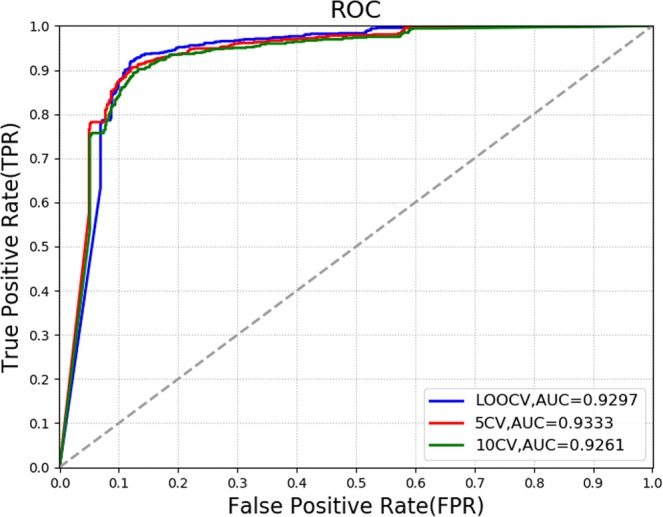
The ROC curves and AUCs of RWRKNN in LOOCV, 5CV and 10CV.

Leave-one-out cross validation (LOOCV), 5-fold cross-validation(5CV) and 10-fold cross-validation(10CV) are utilized to evaluate the prediction performance of our model. For LOOCV, each positive sample is left out in turn as a testing sample, and the other positive samples are used to train the model with the negative samples. Different from the LOOCV, 5CV and 10CV randomly divide the positive samples into 5 equal parts and 10 equal parts respectively, and take out one part of them as testing samples while the rest of samples are regarded as training samples in turn. Next, the predicted scores are sorted in descending order. Further, we draw the receiver operating characteristics (ROC) curve via plotting the true positive rate (TPR) versus the false positive rate (FPR) at different score thresholds. TPR (FPR) refers to the percentage of positive (negative) cases that are correctly identified. Generally, the area under the ROC curve (AUC) is calculated and employed to evaluate the prediction performance. Specifically, the closer the AUC value is to 1, the better the prediction performance. As a result, in LOOCV, RWRKNN achieves an AUC of 0.9297. And concerning 5CV and 10CV, RWRKNN yields the average AUCs of 0.9333 and 0.9261 respectively. The results are shown in the Fig. 2.

### Adjustment of parameters

RWRKNN model involves four parameters: *DA*’s threshold value *α*, *CA*’s threshold value *β*, neighbors’ number *k* and distance metric *p*. The value of *α* and *β* might affect the weighted feature matrixes of circRNAs and diseases. The value of *k* and *p* probably influence KNN’s classification performance. Let *α* and *β* both range between 0.5 and 0.9. Let *k* be an integer value between 1 and 5 and *p* ∈ {1, 2, 3}. As a result, among these four parameters, RWRKNN (*α* = 0.6, *β* = 0.8, *k* = 5 and *p* = 1) gains the highest AUCs of 0.9333 in 5CV as shown in the Supplement. Specifically, *p* = 1 means the Manhattan distance metric.

### Compared with other methods

**Figure 3:**
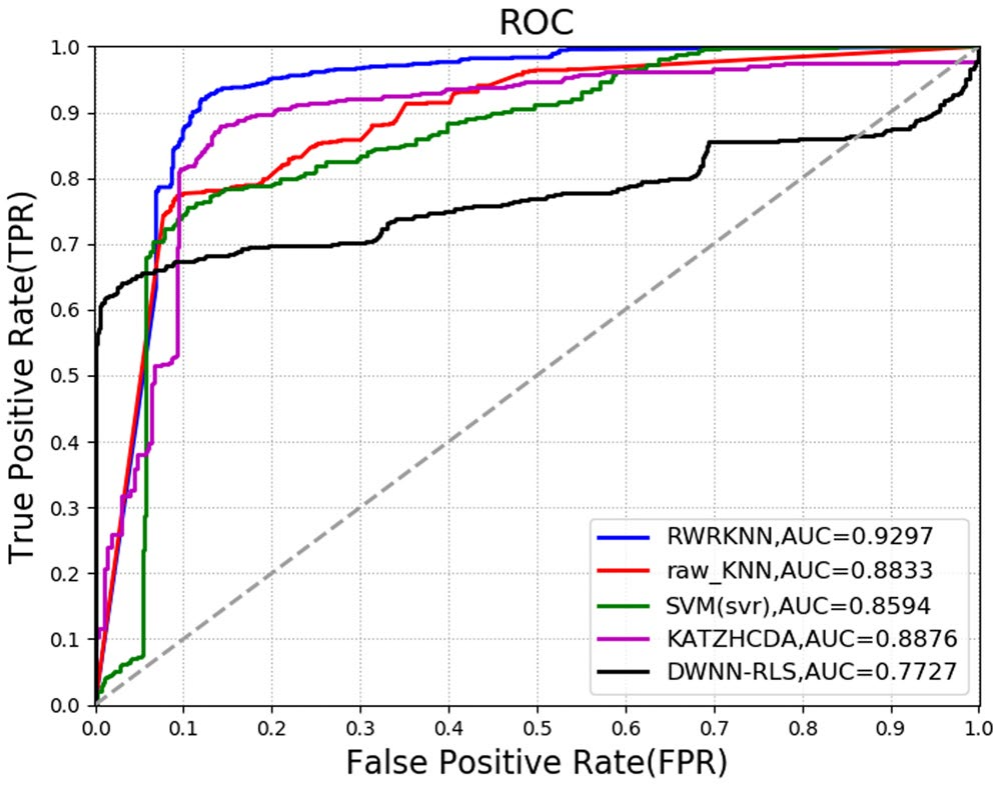
The ROC curves and AUCs of five methods using LOOCV.

**Figure 4:**
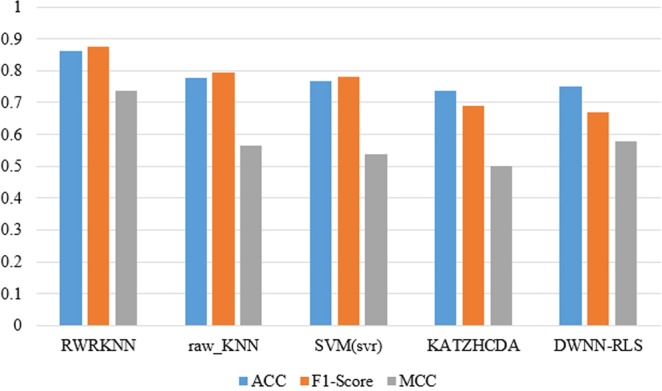
Comparison of five methods in ACC, F1-Score, MCC (LOOCV).

**Figure 5:**
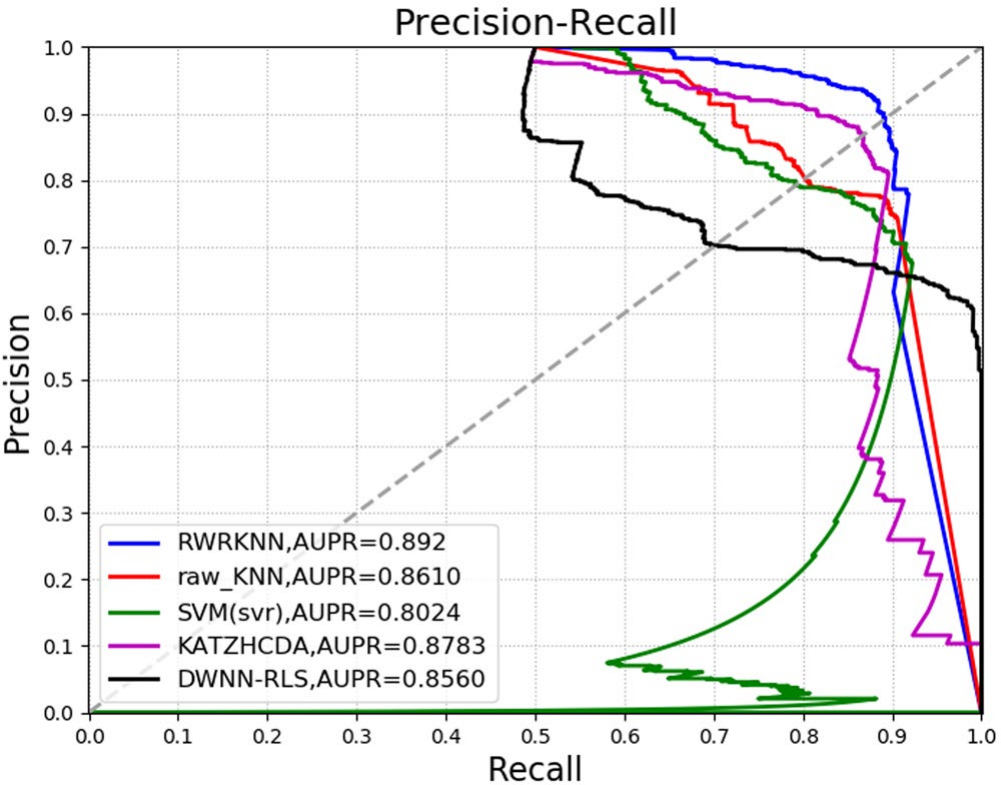
Comparison of five methods in PR curves and AUPRs (LOOCV).

To analyze the performance of RWRKNN model in predicting circRNA-disease associations, RWRKNN (*α* = 0.6, *β* = 0.8, *k* = 5 and *p* = 1) is compared with four methods. Firstly, to show the importance of weighting features, we compared RWRKNN with a model with unweighted features called raw_KNN (*k* = 5, *p* = 1). And in order to highlight the classification performance of KNN, Support Vector Machine (SVM) is compared with our model. In the end, we compare RWRKNN with KATZHCDA^25^ and DWNN-RLS^26^ previously mentioned. The ROC curves of each method using LOOCV are shown in Fig. 3. In addition, we also compared RWRKNN with other four methods in other evaluation criteria (see Fig. 4) including accuracy (ACC), F1-Score, Matthews Correlation Coefficient (MCC). And Precision-Recall (PR) curves and area under the PR curves (AUPRs) are also adopted to reflect the performance of these five methods (see Fig. 5). We can see that RWRKNN gets the satisfactory and optimal performance.

### Case study

To further evaluate the prediction performance of RWRKNN (*α* = 0.6, *β* = 0.8, *k* = 5 and *p* = 1), we also carry out case studies on three common diseases, *i.e*., breast cancer, bladder cancer and colorectal cancer. Breast cancer is one of the most common cancer affecting women, and its incidence and mortality rates are expected to increase significantly the next years^34^. Bladder cancer is a kind of cancer with high incidence, morbidity and mortality^35^. Colorectal cancer is also one of the most common cancers worldwide^36^. However, the complex biology of the three types of diseases remains uncertain and unexplored. Therefore, it is necessary to explore the biological characteristics of these diseases by using computational methods. In this work, all known associations between the investigated disease and circRNAs are assumed to be unknown. Through the calculation of the model, the circRNAs with the top 10 scores are selected among all the predicted associations between the investigated disease and circRNAs. Through searching related literatures or databases, some circRNAs are confirmed to be related to the investigated disease. The results of the case studies of these diseases (breast cancer, bladder cancer and the colorectal cancer) are shown in Tables 1, 2 and 3, respectively.

**Table 1:** Candidate circRNAs of breast cancer.

Rank	CircRNA name	Evidences	Rank	CircRNA name	Evidences
1	hsa_circ_005239	PMID:29037220	6	hsa_circ_0108942	PMID:29045858
2	hsa_circ_0007534	PMID:30139516	7	hsa_circ_0001946	PMID:28049499
3	hsa_circ_0001982	PMID:28933584	8	hsa_circ_0006528	PMID:30520151
4	circRNA-000911	PMID:29431182	9	hsa_circ_0003575	unconfirmed
5	hsa_circ_0001785	PMID:29045858	10	circDENND4C	PMID:31488193

**Table 2:** Candidate circRNAs of bladder cancer.

Rank	CircRNA name	Evidences	Rank	CircRNA name	Evidences
1	hsa_circ_0003221	PMID:29125888	6	hsa_circ_0007158	circRNADisease
2	hsa_circ_0091017	PMID:29151929	7	hsa_circ_0041103	circRNADisease
3	hsa_circ_0000284	circRNADisease	8	hsa_circ_0008732	unconfirmed
4	hsa_circ_0002768	circRNADisease	9	hsa_circ_0005941	unconfirmed
5	hsa_circ_0058058	unconfirmed	10	hsa_circ_0002024	PMID:30972190

**Table 3:** Candidate circRNAs of colorectal cancer.

Rank	CircRNA name	Evidences	Rank	CircRNA name	Evidences
1	hsa_circ_0007534	PMID:29364478	6	hsa_circ_0020397	PMID:28707774
2	hsa_circ_0001649	PMID:29421663	7	circ-BANP	PMID:28103507
3	hsa_circ_0014717	PMID:29571246	8	hsa_circ_0000069	PMID:28003761
4	hsa_circ_0000567	PMID:29333615	9	hsa_circRNA_104700	PMID:28349836
5	circRNA0003906	PMID:29123417	10	hsa_circRNA_103809	PMID:30249393

## Conclusion

At circRNA level, identifying unknown associations of circRNA-disease get crucial for the study of biomarkers for disease diagnosis. In this study, a computational method (RWRKNN) is proposed, which integrates RWR and KNN regression. The existing circRNA-disease association from CircR2Disease is used to assign labels to circRNA-disease pairs. In view of constructing feature of circRNA-disease pairs and circRNA-circRNA associations *CA* and disease-disease associations *DA*, we take use of circRNA-disease associations, GIP kernel similarities of circRNAs and diseases, circRNA functional similarity and disease semantic similarity. For every circRNA (disease), we complement RWR on the constructed *CA* (*DA*) matrix to obtain affinity scores, which are employed to weight the features of circRNAs (diseases). After obtaining the global feature vectors of circRNAs (diseases), KNN regression model could output the possibility of inquired circRNA-disease association pairs. In addition, both multiple performance evaluation criteria and case studies on breast cancer, bladder cancer and colorectal cancer have illustrated the reliable prediction ability of RWRKNN. However, RWRKNN also has limitations. It relies on prior information about circRNAs and diseases. Therefore, it is slightly inadequate in uncovering the relationship between new diseases and new circRNAs.

## Materials and Methods

### Human circRNA-disease associations

To acquire circRNA-disease associations verified by biological experiments, we download the circRNA-disease associations from circR2Disease database (http://bioinfo.snnu.edu.cn/CircR2Disease/)^22^. CircR2Disease provides association information between circRNAs and diseases supported by experiments, including 725 circRNA-disease associations between 661 circRNAs and 100 diseases. In this study, we extract all circRNA and disease associations in the database and then construct a matrix *A* to reflect the adjacency associations of circRNA-disease. If a disease *i* has been confirmed to have an association with a circRNA *j*, *A*(*i*, *j*) = 1, otherwise *A*(*i*, *j*) = 0. The dimension of *A* is *N*_*c*_ × *N*_*d*_, where *N*_*c*_ and *N*_*d*_ represent the number of the known circRNAs and the known diseases, respectively.

### Disease similarity

The semantic similarity between diseases is calculated based on DAG (directed acyclic graph) topology. To be specific, the DAG of a disease *d* can be defined as DAG(*d*) = (*d*, T(*d*), E(*d*)), where T(*d*) is an ancestor set of disease *d* and E(*d*) includes the corresponding edges. According to Eqs. (1) and (2), the semantic value DSV(*d*) of disease *d* can be obtained^37^.

1 $${D}_{d}(t)=\{\begin{array}{rr}1, & if\,t=d\\ {\rm{\max }}\,\{\Delta \ast {D}_{d}(t^{\prime} )|t^{\prime} \in {\rm{children}}\,{\rm{of}}\,t\}, & if\,t\ne d\end{array}$$

2 $$DSV(d)={\sum }_{t\in T(d)}{D}_{d}(t)$$

where the disease *t* ∈ T(*d*), ∆ (∆ = 0.5) is semantic contribution decay factor, and *D*_*d*_(*t*) denotes the contribution of ancestor node *t* to *d*. Next, the semantic similarity between *d*_*i*_ and *d*_*j*_ can be calculated as follows:

3 $$DSS({d}_{i},{d}_{j})=\frac{{\sum }_{t\in T({d}_{i})\cap T({d}_{j})}({D}_{{d}_{i}}(t)+{D}_{{d}_{j}}(t))}{DSV({d}_{i})+DSV({d}_{j})}$$

In the end, the semantic similarity matrix of diseases *DSS* is constructed.

GIP kernel similarity is calculated based on the topological structure of the association network of biological information nodes^38^. An assumption supporting this approach is that more similar diseases tend to be associated with the similar circRNAs^38^. Disease GIP kernel similarity is calculated with the known circRNA-disease associations, which is obtained according to the Eq. (4).

4 $$DGS(i,j)=\exp (-{\sigma }_{d}{\Vert I{P}_{d(i)}-I{P}_{d(j)}\Vert }^{2})$$

where *IP*_*d(i)*_ represents the interaction profile of disease *i* as a binary vector reflecting whether disease *i* is associated with each circRNA or not. *DGS*(*i*, *j*) is the GIP kernel similarity between disease *i* and disease *j. σ*_*d*_ is influential in tuning the kernel bandwidth calculated by the Eq. (5).

5 $${\sigma }_{d}={\sigma }_{d}^{\ast }/(\frac{1}{{N}_{d}}\mathop{\sum }\limits_{i=1}^{{N}_{d}}{\Vert I{P}_{d(i)}\Vert }^{2})$$

where *N*_*d*_ is the number of all diseases, and ${\sigma }_{d}^{\ast }$ is set to 1 as the initial value following the previous study^38^.

In order to make full use of the disease semantic similarity and the disease GIP kernel similarity, we construct a new disease similarity matrix *SD* (See Fig. 1b) by integrating *DSS* and *DGS* based on the Eq. (6).

6 $$SD(d(i),d(j))=\{\begin{array}{cc}\frac{DSS(d(i),d(j))+DGS(d(i),d(i))}{2} & if\,DSS(d(i),d(j))\ne 0\\ DGS(d(i),d(j)) & otherwise\end{array}$$

### CircRNA similarity

Similar to the calculation method of disease GIP kernel similarity, we use the Eq. (7) to calculate circRNA GIP kernel similarity.

7 $$CGS(i,j)=\exp (\,-\,{\sigma }_{c}{\Vert I{P}_{{\rm{c}}(i)}-I{P}_{{\rm{c}}(j)}\Vert }^{2})$$

where *IP*_*c*(*i*)_ represents the interaction profile of circRNA *i* as a binary vector reflecting whether circRNA *i* is associated with each disease or not. *CGS*(*i*, *j*) is the GIP kernel similarity between circRNA *i* and circRNA *j. σ*_*c*_ is influential in tuning the kernel bandwidth calculated by the Eq. (8).

8 $${\sigma }_{c}={\sigma }_{c}^{\ast }/(\frac{1}{{N}_{c}}\mathop{\sum }\limits_{i=1}^{{N}_{c}}{\Vert I{P}_{{\rm{c}}(i)}\Vert }^{2})$$

where *N*_*c*_ is the number of all circRNAs, and ${\sigma }_{c}^{\ast }$ is set to 1 as the initial value following the previous study^38^.

We adopt a similar method to Wang’s method^37^ for calculating circRNA functional similarity to improve the accuracy of the calculation model. To be specific, the functional similarity score between a circRNA *U* and a circRNA *V* is obtained by calculating the semantic similarity between the two groups of circRNA-related diseases. First, let d*x* be any given disease, and D*y* be a group of diseases defined as *Dy* = {*dy*_1_, *dy*_2_, *dy*_3_, …, *dy*_*r*_}. Then, the semantic similarity between d*x* and D*y* can be calculated as follows:

9 $$SS(dx,Dy)=\mathop{{\rm{\max }}}\limits_{1\le i\le r}(DSS(dx,d{y}_{i}))$$

Second, the functional similarity between circRNA *U* and circRNA *V* can be calculated as follows:

10 $$CFS(U,{\rm{V}})=\frac{{\sum }_{1\le i\le |Du|}SS(D{u}_{i},Dv)+{\sum }_{1\le j\le |Dv|}SS(D{v}_{j},Du)}{|Du|+|Dv|}$$

where *Du* is a group of circRNA *U*-related diseases and *Dv* is another group of circRNA *V*-related diseases. D*u*_*i*_ ∈ D*u* and D*v*_*j*_ ∈ D*v*. In the end, circRNA functional similarity matrix is constructed, which is symmetric and has all 1 s on its diagonal. *CFS*(*i*, *j*) represents the functional similarity between circRNA *i* and circRNA *j*.

Similar to the method of disease similarity integration, circRNA functional similarity and circRNA GIP kernel similarity are integrated to constitute a new circRNA similarity matrix *SC* (See Fig. 1a) based on the Eq. (11).

11 $$SC(c(i),c(j))=\{\begin{array}{cc}\frac{CFS(c(i),c(j))+CGS(c(i),c(i))}{2} & if\,CFS(c(i),c(j))\ne 0\\ CGS(c(i),c(j)) & otherwise\end{array}$$

### Human disease-disease associations

In order to get the disease association adjacency matrix *DA*, a threshold value α is set for the integrated disease similarity *SD* as shown in Eq. (12). If the similarity value is greater or equal to α, the corresponding position in *DA* has a value of 1, otherwise 0.

12 $$DA(i,j)=\{\begin{array}{cc}1 & if\,SD(i,\,j)\ge \alpha \\ 0 & otherwise\,\end{array}$$

### Human circRNA- circRNA associations

In order to get the circRNA association adjacency matrix *CA*, a threshold value *β* is set for the integrated circRNA similarity *SC* as shown in Eq. (13). If the similarity value is greater or equal to *β*, the corresponding position in *CA* has a value of 1, otherwise 0.

13 $$CA(i,j)=\{\begin{array}{cc}1 & if\,SC(i,\,j)\ge \beta \\ 0 & otherwise\,\end{array}$$

### RWRKNN

After having constructed four matrixes, *i.e*., the integrated disease similarity matrix, the integrated circRNA similarity matrix, disease-disease association matrix and circRNA-circRNA association matrix, RWRKNN will do the following three steps, *i.e*., RWR for every circRNA and disease (Fig. 1c,d), feature weighting (Fig. 1e,f) and training KNN model (Fig. 1g).

Considering the input requirements of the KNN regression model, we transform the features of circRNA-disease pairs into vectors. Firstly, for diseases, we take each row of the integrated disease similarity *SD* as the feature vector of diseases with 100 dimensions. Similarly, with respect to circRNAs, we take each row of the integrated circRNA similarity *SC* as the feature vector of circRNAs with 661 dimensions.

To make predictions of circRNA-disease associations from a global network perspective, we could obtain affinity scores between a circRNA (disease) node and all circRNA (disease) nodes using the RWR algorithm on the *CA* (*DA*) network. RWR estimates affinity level (affinity score) between two nodes by repeatedly exploring the overall structure of a network. Starting at a seed node, the random walker diffuses its resources by (1) moving to a neighbor node with probability 1-*c* and (2) restarting from the seed node with restarting probability *c*. This process is iterated repeatedly until all nodes are traversed. At this time, the probability vector obtained contains the affinity scores of all nodes and the seed node. The affinity scores of all nodes during each step are represented by the Eq. (14).

14 $$p=(1-c)Wp+cq$$

where *q* is the starting vector whose seed node is set to 1 while the others are set to 0, and *W* is the normalized adjacency matrix, and *p* would finally reach a steady state after multiple iterations, and *c* is set to 0.7 according to Park *et al*.^39^’s work. Consequently, by multiplying the adjacency matrix, it diffuses its resources throughout the network. By the iteration of the *p*_*i*_ value, *i.e*. the result of RWR for the seed node *i*, the affinity score matrix *F* could be obtained, whose element *F*_*ij*_ refers to how closely node *j* is connected to seed node *i*. Finally, the (*N*_*c*_ × *N*_*c*_) circRNA affinity score matrix *F*^*c*^ and the (*N*_*d*_ × *N*_*d*_) disease affinity score matrix *F*^*d*^ are constructed, where *N*_*c*_ is the number of circRNAs and *N*_*d*_ is the number of diseases.

Next, we utilize the affinity scores to weight the circRNA and disease features. As regards the circRNA features, they are weighted using the Eq. (15).

15 $$WSC(c(i))={F}_{i}^{{c}^{T}}\times SC(c(i))$$

where ${F}_{i}^{c}$ means the affinity score of circRNA *c*(*i*), which is a row vector. ${F}_{i}^{{c}^{T}}$ is the transpose of ${F}_{i}^{c}$. And *SC*(*c*(*i*)) denotes the integrated similarity of circRNA *c*(*i*), which is also a row vector. *WSC* is the weighted feature matrix of circRNAs and *WSC*(*c*(*i*)) represents the weighted feature of circRNA *c*(*i*).

In the case of the disease features, Eq. (16) is used.

16 $$WSD(d(i))={F}_{i}^{{d}^{T}}\times SD(d(i))$$

where ${F}_{i}^{d}$ means the affinity score of disease *d*(*j*), which is a row vector. ${F}_{i}^{{d}^{T}}$ is the transpose of ${F}_{i}^{d}$. And *SC*(*d*(*j*)) denotes the integrated similarity of disease *d*(*j*), which is also a row vector. *WSD*(*d*(*j*)) represents the weighted feature of disease *d*(*j*) and *WSD* is the weighted feature matrix of diseases.

In the whole, the features of circRNAs and diseases are weighted by means of adding each feature of all nodes to a certain seed node via affinity scores from the RWR. The weighting can be conducted by multiplying feature matrix by affinity score matrix as depicted in Fig. 1e,f.

With the weighted features of the circRNAs and diseases, we link each feature vector of diseases and circRNAs together to compose a 761-dimensional feature vector for each circRNA-disease pair as the input of KNN regressor model. To train the KNN regressor model, we prepare positive samples and negative samples. The known circRNA-disease association pairs are used as positive samples. To get negative samples, the following steps are taken: (1) A circRNA *i* is selected at first, and then (2) calculate the number *nd*_*i*_ of diseases associated with the circRNA *i*. (3) Next, select *nd*_*i*_ diseases unassociated to the circRNA *i*. (4) Until all circRNAs are traversed, we end up with the same number of negative samples as positive samples. In RWRKNN model, the KNN regression could find *k* neighbors closest to a certain circRNA-disease pair based on the Minkowski distance metric (defined as Eq. (17)), which is a set of distance definitions.

17 $$d(x,y)={(\mathop{\sum }\limits_{i=1}^{n}|{a}_{i}-{{b}_{i}|}^{p})}^{\frac{1}{p}}$$

Here, different values of *p* represent different distance metrics for calculating the distance between vectors *a* and *b* with *n*-dimension, and we set *p* = 1, which represents the Manhattan distance is used as a metric between vectors. In addition, considering the closer neighbors should have more weight, we take the inverse of the distance as the weight.

## Supplementary information

Supplementary information

## Data Availability

Data can be obtained by sending an email to the corresponding author upon reasonable request.
