# Supplementary material for: Integrating random walk with restart and k-Nearest Neighbor to identify novel circRNA-disease association
Source: Sci Rep. 2020 Feb 6;10:1943. doi: 10.1038/s41598-020-59040-0 (PMC7005057; doi:10.1038/s41598-020-59040-0)
Supplement: Supplementary file 1 — Supplementary information [file 41598_2020_59040_MOESM1_ESM.pdf]

Integrating random walk with restart and k-Nearest Neighbor to identify novel circRNA-disease association

Xiujuan Lei *, Chen Bian

The results of parameter adjustment：

α	β	k	p	AUC (descending sort)
0.6	0.8	5	1	0.933294649
0.6	0.8	5	2	0.929868252
0.5	0.9	5	1	0.929398335
0.5	0.8	5	1	0.92860975
0.7	0.5	5	1	0.927351249
0.9	0.7	4	1	0.92669679
0.8	0.6	5	2	0.92662069
0.7	0.7	4	1	0.926322949
0.5	0.9	5	2	0.925756956
0.6	0.9	5	1	0.925652319
0.6	0.7	5	2	0.925243282
0.8	0.6	5	1	0.923945779
0.5	0.5	5	1	0.92390868
0.7	0.9	5	2	0.922800476
0.5	0.7	5	3	0.922390488
0.5	0.8	4	1	0.922355291
0.7	0.8	5	2	0.922134602
0.7	0.7	5	2	0.921773127
0.5	0.7	5	2	0.921748395
0.5	0.5	4	2	0.921688466
0.5	0.7	5	1	0.92165327
0.6	0.7	4	2	0.921504875
0.9	0.9	5	1	0.921411653
0.8	0.9	5	2	0.92111962
0.8	0.9	5	1	0.921000713
0.6	0.6	4	1	0.92082283
0.5	0.8	4	3	0.920799049
0.6	0.5	4	1	0.920578359
0.7	0.8	5	1	0.920387158
0.9	0.5	5	3	0.920226397
0.8	0.8	4	2	0.920184542
0.5	0.6	4	1	0.920116052
0.8	0.8	4	1	0.92
0.6	0.9	5	2	0.91997717
0.7	0.5	5	3	0.919962901
0.6	0.6	4	2	0.919803092
0.8	0.9	4	2	0.919731748
0.6	0.6	5	1	0.91939025
0.5	0.6	5	1	0.919294174
0.8	0.7	4	2	0.919257075
0.7	0.8	4	1	0.919015458

0.7	0.6	5	1	0.918919382
0.8	0.5	5	1	0.918777646
0.8	0.8	5	3	0.918713912
0.9	0.6	5	2	0.918588347
0.5	0.8	5	3	0.918487515
0.5	0.5	5	3	0.918116528
0.9	0.5	4	1	0.918107015
0.6	0.6	5	2	0.918049941
0.9	0.6	4	3	0.917758859
0.6	0.8	3	2	0.917611415
0.6	0.5	5	3	0.917579073
0.6	0.9	4	1	0.917536266
0.5	0.7	3	3	0.9175239
0.8	0.8	5	2	0.917434483
0.7	0.7	5	1	0.917399287
0.9	0.6	5	1	0.917392628
0.7	0.9	4	3	0.9170283
0.7	0.7	5	3	0.916980737
0.5	0.5	4	3	0.916930321
0.5	0.5	5	2	0.916927467
0.9	0.5	5	2	0.91682283
0.7	0.6	4	3	0.916682045
0.6	0.8	3	1	0.916538407
0.5	0.8	5	2	0.916448989
0.6	0.5	5	2	0.916433769
0.5	0.8	3	2	0.916423306
0.9	0.8	5	3	0.916386207
0.9	0.7	5	2	0.916343401
0.5	0.8	4	2	0.916174078
0.5	0.7	4	2	0.916151249
0.8	0.9	4	1	0.916079905
0.6	0.8	4	1	0.916069441
0.8	0.7	5	3	0.915948633
0.7	0.8	4	3	0.915770749
0.5	0.6	5	3	0.915613793
0.9	0.9	4	2	0.915285612
0.9	0.8	5	2	0.915239952
0.5	0.7	4	1	0.914952675
0.5	0.8	3	1	0.91494126
0.8	0.8	5	1	0.914935553
0.8	0.9	5	3	0.914901308
0.9	0.5	5	1	0.914827111
0.7	0.5	5	2	0.91464352
0.6	0.9	5	3	0.914492271
0.9	0.6	5	3	0.91448371
0.6	0.6	5	3	0.914458026
0.7	0.7	4	3	0.914409512
0.7	0.6	4	2	0.914119382

0.8	0.5	3	3	0.914112723
0.5	0.9	5	3	0.914050892
0.8	0.6	4	1	0.914016647
0.9	0.9	3	1	0.914010939
0.5	0.6	5	2	0.914002378
0.9	0.7	5	1	0.913995719
0.6	0.6	3	2	0.913945303
0.9	0.5	4	2	0.913757907
0.9	0.7	5	3	0.91372176
0.5	0.5	3	3	0.913686564
0.8	0.5	5	3	0.913456361
0.6	0.5	5	1	0.913410702
0.7	0.9	5	1	0.9133717
0.9	0.8	5	1	0.913107253
0.7	0.9	5	3	0.913015933
0.6	0.9	4	2	0.912928419
0.9	0.5	4	3	0.912759096
0.6	0.7	5	3	0.912749584
0.6	0.5	4	3	0.912700119
0.6	0.7	4	1	0.912612604
0.6	0.6	4	3	0.912555529
0.9	0.9	4	3	0.91250321
0.6	0.6	3	3	0.91206849
0.6	0.5	3	2	0.91200761
0.9	0.6	4	2	0.911709869
0.8	0.5	4	1	0.911651843
0.8	0.7	5	1	0.911649941
0.6	0.8	5	3	0.911649941
0.5	0.5	3	1	0.911539596
0.5	0.9	3	3	0.911392152
0.6	0.6	3	1	0.911318906
0.5	0.9	3	1	0.911197146
0.6	0.5	4	2	0.911027824
0.8	0.5	4	2	0.91101736
0.7	0.7	4	2	0.910935553
0.6	0.9	4	3	0.910616885
0.7	0.8	5	3	0.910602616
0.9	0.8	4	2	0.910592152
0.9	0.7	4	2	0.910578835
0.5	0.9	4	3	0.910502735
0.5	0.9	4	1	0.910473246
0.7	0.8	3	2	0.910465636
0.9	0.9	5	3	0.910431391
0.5	0.7	4	3	0.910416171
0.6	0.5	3	3	0.910011891
0.8	0.5	5	2	0.909975743
0.5	0.6	4	2	0.909867301
0.5	0.6	4	3	0.909799762

0.5	0.6	3	1	0.909724614
0.7	0.5	4	1	0.909693222
0.7	0.6	5	3	0.909542925
0.7	0.9	4	1	0.909375505
0.6	0.7	5	1	0.909283234
0.7	0.6	5	2	0.909248038
0.8	0.7	4	1	0.909141498
0.8	0.7	3	1	0.909062545
0.8	0.9	4	3	0.908972176
0.5	0.5	4	1	0.908885612
0.5	0.9	3	2	0.908871344
0.9	0.6	3	3	0.908645898
0.5	0.6	3	3	0.908430916
0.5	0.5	3	2	0.908316766
0.7	0.5	3	3	0.907970511
0.8	0.5	4	3	0.907963853
0.7	0.9	3	1	0.907852556
0.7	0.8	4	2	0.907839239
0.6	0.9	3	2	0.907765042
0.9	0.9	4	1	0.907631867
0.6	0.8	4	3	0.907474911
0.9	0.8	4	1	0.907452081
0.9	0.9	5	2	0.907128656
0.8	0.8	4	3	0.907073484
0.8	0.7	5	2	0.907026873
0.8	0.6	4	3	0.906755767
0.8	0.6	3	2	0.906288704
0.7	0.5	4	3	0.906229727
0.6	0.9	3	1	0.9060956
0.8	0.6	3	1	0.906037574
0.5	0.7	3	2	0.905953864
0.8	0.7	3	3	0.905944352
0.5	0.7	3	1	0.905931034
0.9	0.7	4	3	0.905756005
0.9	0.8	3	2	0.905258502
0.8	0.8	3	1	0.905188109
0.5	0.8	3	3	0.905053032
0.6	0.7	3	1	0.905017836
0.5	0.9	4	2	0.904861831
0.8	0.7	4	3	0.90478478
0.8	0.5	3	1	0.904336742
0.6	0.5	3	1	0.904331986
0.9	0.8	4	3	0.904314863
0.9	0.6	3	2	0.904249227
0.9	0.9	3	2	0.904206421
0.7	0.9	4	2	0.90406088
0.8	0.6	4	2	0.903978121
0.9	0.5	3	3	0.90381736

0.8	0.8	3	3	0.903765993
0.6	0.9	2	1	0.903751724
0.7	0.5	3	2	0.903574792
0.7	0.6	3	1	0.903558621
0.9	0.9	3	3	0.903388347
0.8	0.7	3	2	0.90320761
0.9	0.5	3	2	0.902787158
0.9	0.6	4	1	0.902732937
0.7	0.5	4	2	0.902657788
0.5	0.6	3	2	0.902614031
0.5	0.8	2	3	0.902248751
0.8	0.6	5	3	0.902228775
0.7	0.7	3	1	0.901821641
0.7	0.6	3	3	0.90153912
0.7	0.7	3	3	0.901415458
0.7	0.6	3	2	0.901314625
0.8	0.6	3	3	0.901192866
0.8	0.9	3	2	0.90076956
0.6	0.7	3	2	0.900666825
0.7	0.8	3	1	0.900544114
0.5	0.7	2	2	0.900474673
0.5	0.9	2	2	0.900458502
0.8	0.9	3	3	0.900313912
0.7	0.6	4	1	0.900293936
0.9	0.8	3	1	0.900214031
0.5	0.9	2	1	0.900046611
0.6	0.7	3	3	0.899873484
0.9	0.7	2	2	0.899842093
0.9	0.7	3	2	0.899775505
0.8	0.9	3	1	0.899753627
0.9	0.7	3	3	0.89972604
0.5	0.7	2	1	0.899708918
0.9	0.6	2	1	0.899643282
0.6	0.7	4	3	0.899575743
0.9	0.6	3	1	0.89939025
0.5	0.9	2	3	0.899326516
0.5	0.8	2	1	0.899096314
0.7	0.7	3	2	0.898858502
0.6	0.8	3	3	0.898632105
0.5	0.5	2	1	0.898265874
0.6	0.6	2	1	0.897568609
0.9	0.7	3	1	0.897360285
0.5	0.6	2	1	0.897307015
0.7	0.9	2	1	0.897255648
0.7	0.8	2	1	0.896960761
0.7	0.9	3	3	0.896785731
0.7	0.5	3	1	0.896580262
0.6	0.8	4	2	0.896530797

0.6	0.5	2	3	0.896482283
0.6	0.7	2	1	0.89623591
0.8	0.5	3	2	0.896070392
0.8	0.9	2	3	0.895880143
0.6	0.7	2	2	0.895818312
0.5	0.5	2	2	0.895643282
0.8	0.7	2	2	0.895436861
0.7	0.9	2	3	0.895214269
0.7	0.9	2	2	0.895128656
0.7	0.9	3	2	0.894977408
0.7	0.7	2	1	0.894601665
0.9	0.9	2	2	0.894242093
0.5	0.8	2	2	0.894044233
0.8	0.8	3	2	0.894044233
0.6	0.9	3	3	0.894011891
0.9	0.7	2	1	0.893826397
0.7	0.8	3	3	0.893807372
0.9	0.5	2	2	0.893677051
0.8	0.8	2	2	0.893421165
0.8	0.6	2	1	0.893350773
0.8	0.5	2	1	0.893277527
0.8	0.5	2	2	0.893274673
0.5	0.7	2	3	0.893224257
0.9	0.8	3	3	0.893223306
0.7	0.6	2	3	0.892904637
0.6	0.5	2	2	0.89279239
0.8	0.9	2	2	0.892788585
0.5	0.5	2	3	0.89225874
0.7	0.6	2	2	0.892246373
0.5	0.6	2	2	0.891785969
0.9	0.5	3	1	0.891715577
0.8	0.7	2	3	0.891427348
0.6	0.6	2	2	0.890648276
0.7	0.8	2	3	0.890477051
0.7	0.5	2	1	0.890201189
0.6	0.7	2	3	0.890003329
0.8	0.8	2	1	0.889892033
0.6	0.8	2	2	0.889370749
0.6	0.9	2	3	0.889337455
0.6	0.9	2	2	0.889069203
0.6	0.8	2	1	0.888821879
0.8	0.7	2	1	0.888498454
0.7	0.6	2	1	0.888194055
0.8	0.9	2	1	0.88818264
0.6	0.5	2	1	0.887881094
0.8	0.6	2	3	0.887736504
0.5	0.6	2	3	0.887649941
0.9	0.9	2	1	0.887523424

0.9	0.7	2	3	0.887375981
0.6	0.8	1	1	0.886896552
0.9	0.5	2	1	0.886611177
0.8	0.9	1	2	0.886206897
0.9	0.9	2	3	0.88614126
0.7	0.5	2	2	0.88591201
0.9	0.8	2	1	0.885837812
0.7	0.8	2	2	0.885656124
0.5	0.9	1	1	0.885517241
0.9	0.8	2	2	0.88548585
0.6	0.8	2	3	0.885082521
0.5	0.8	1	2	0.884827586
0.5	0.5	1	3	0.884137931
0.5	0.8	1	1	0.884137931
0.5	0.9	1	2	0.884137931
0.6	0.8	1	2	0.883448276
0.8	0.6	2	2	0.882493222
0.8	0.5	2	3	0.882441855
0.7	0.7	2	3	0.882273484
0.6	0.7	1	1	0.882068966
0.8	0.9	1	1	0.882068966
0.9	0.6	1	1	0.882068966
0.9	0.7	1	1	0.88137931
0.5	0.5	1	2	0.880689655
0.5	0.6	1	1	0.880689655
0.5	0.6	1	3	0.880689655
0.9	0.9	1	2	0.880689655
0.9	0.5	2	3	0.88014459
0.5	0.8	1	3	0.88
0.5	0.9	1	3	0.88
0.5	0.7	1	3	0.88
0.8	0.8	2	3	0.879537693
0.9	0.6	2	2	0.879396908
0.9	0.6	2	3	0.879334126
0.5	0.6	1	2	0.879310345
0.9	0.5	1	1	0.879310345
0.6	0.9	1	1	0.87862069
0.6	0.9	1	2	0.87862069
0.7	0.5	1	1	0.87862069
0.8	0.6	1	1	0.87862069
0.8	0.8	1	2	0.87862069
0.6	0.6	2	3	0.878523662
0.6	0.6	1	1	0.877931034
0.7	0.6	1	1	0.877931034
0.7	0.7	2	2	0.877340309
0.6	0.7	1	2	0.877241379
0.7	0.7	1	2	0.876551724
0.9	0.5	1	2	0.876551724

0.7	0.9	1	2	0.876551724
0.9	0.8	2	3	0.876415696
0.5	0.7	1	2	0.875862069
0.7	0.5	1	2	0.875862069
0.8	0.7	1	2	0.875862069
0.5	0.5	1	1	0.875172414
0.5	0.7	1	1	0.875172414
0.7	0.7	1	3	0.875172414
0.6	0.9	1	3	0.874482759
0.7	0.8	1	2	0.873793103
0.8	0.5	1	1	0.873793103
0.9	0.9	1	1	0.873793103
0.9	0.6	1	2	0.873103448
0.6	0.5	1	1	0.873103448
0.8	0.7	1	1	0.872413793
0.8	0.8	1	3	0.872413793
0.7	0.6	1	3	0.871724138
0.8	0.8	1	1	0.871724138
0.6	0.5	1	2	0.871034483
0.9	0.8	1	1	0.871034483
0.6	0.5	1	3	0.870344828
0.7	0.5	2	3	0.869878716
0.7	0.7	1	1	0.868965517
0.7	0.8	1	1	0.868965517
0.7	0.8	1	3	0.868965517
0.8	0.5	1	2	0.868965517
0.7	0.5	1	3	0.868275862
0.7	0.9	1	1	0.868275862
0.7	0.9	1	3	0.868275862
0.8	0.6	1	2	0.868275862
0.8	0.6	1	3	0.868275862
0.9	0.7	1	2	0.868275862
0.9	0.7	1	3	0.868275862
0.6	0.8	1	3	0.867586207
0.9	0.5	1	3	0.867586207
0.6	0.6	1	2	0.866206897
0.8	0.7	1	3	0.865517241
0.8	0.9	1	3	0.865517241
0.9	0.8	1	2	0.864827586
0.7	0.6	1	2	0.864137931
0.6	0.7	1	3	0.862758621
0.9	0.8	1	3	0.862758621
0.6	0.6	1	3	0.86137931
0.8	0.5	1	3	0.86137931
0.9	0.6	1	3	0.860689655
0.9	0.9	1	3	0.860689655
